# Singapore Housing Index and prevalence of serious bacterial infections among febrile infants

**DOI:** 10.3389/fped.2026.1716413

**Published:** 2026-03-02

**Authors:** Annisa Dewi Utami Rakun, Jin Wee Lee, Sarah Hui Wen Yao, Lena Wong, Rupini Piragasam, Gene Yong-Kwang Ong, Zi Xean Khoo, Andrew Fu Wah Ho, Sashikumar Ganapathy, Shu-Ling Chong

**Affiliations:** 1 Department of Children’s Emergency, KK Women’s and Children’s Hospital, Singapore, Singapore; 2 Duke-NUS Medical School, Singapore, Singapore; 3 Department of Paediatric Medicine, KK Women’s and Children’s Hospital, Singapore, Singapore; 4 KK Research Centre, KK Women’s and Children’s Hospital, Singapore, Singapore; 5 Emergency Medicine Academic Clinical Programme, SingHealth Duke-NUS, Singapore, Singapore; 6 Paediatrics Academic Clinical Programme, SingHealth Duke-NUS, Singapore, Singapore

**Keywords:** febrile infants, serious bacterial infection (SBI), Singapore, Singapore Housing Index, socioeconomic factors

## Abstract

**Background and objective:**

This study aimed to examine the relationship between the Singapore Housing Index (SHI) and presence of serious bacterial infections (SBIs) among young febrile infants.

**Methods:**

A secondary analysis was conducted on infants ≤3 months old, who presented to a paediatric Emergency Department (ED) with temperature ≥38°C between December 2017 and 2021. SHI was categorised into low, medium and high groups. The primary outcome was presence of SBIs. Secondary outcome was a composite of the need for resuscitation, and/or need for high acuity care. We performed multivariable logistic regression to study if SHI was independently associated with SBIs and SBI outcomes.

**Results:**

Among 1,001 infants, the median age was 32 days (interquartile range IQR 10–60), and 176 infants (17.6%) were diagnosed with SBIs. The SBI rates among low, medium, and high SHI groups were 12.6% (13/103), 17.5% (115/658), and 20% (48/240) respectively (*p* = 0.256). After adjusting for male sex, neonate status, ethnicity, and late prematurity, neither high (aOR 1.714, 95% CI 0.844–3.480, *p* = 0.136) nor medium SHI (aOR 1.572, 95% CI 0.826–2.990, *p* = 0.168) was significantly associated with SBI compared with low SHI. No evidence of association was found between SHI and severe clinical outcomes.

**Conclusions:**

In our study population, young infants from low SHI were not at greater risk for SBIs. Future research should include other measures of social determinants in the understanding of SBI risk in young febrile infants.

## Introduction

Fever is a common clinical presentation to the paediatric emergency department (ED), estimated to account for about 20% of paediatric ED visits (1). Serious bacterial infections (SBIs) (defined as bacteraemia, meningitis, and urinary tract infections) occur in about 10%–15% of febrile infants ≤3 months of age and can potentially lead to death or end organ dysfunction (2–5).

Previous research identified healthcare disparities among febrile infants of different socioeconomic status (SES). For example, a cross-sectional study in Philadelphia, United States of America (USA), found that infants from neighbourhoods with high childhood poverty rates were more than 3-times as likely to develop SBIs compared to those from more affluent areas (6). Additionally, a joint study from Bangladesh, India and Pakistan identified low SES to be a risk factor for SBIs for infants two months and younger (7). In New Zealand, the most socioeconomically deprived individuals had a higher risk of hospitalisation for severe infectious diseases across all age groups (8). These findings suggest that socioeconomic status may be an influential factor on the prevalence and severity of SBIs and further research is important to drive changes in policy.

In Singapore, public agencies have proactively engaged disadvantaged families of lower SES to improve health literacy and maternal-child health through community initiatives (9). However, SBI prevalence and outcomes among various SES groups remain unexplored. This study aimed to investigate the association between socioeconomic status and the presence of SBIs, using Singapore Housing Index (SHI) as a surrogate for SES. We hypothesised that infants with higher SHI, indicating higher SES, would have a lower incidence of SBIs compared to lower SHI. In addition, we aimed to determine whether SHI influenced SBI outcomes. We hypothesised that infants with higher SHI would have better clinical outcomes, including shorter hospital stay and reduced need for high resource medical intervention such as fluid bolus resuscitation, inotropic support, emergent intravenous antibiotics, ventilator support, or admission to the high dependency or intensive care unit care. As a corollary, the findings from this study can guide public health policies to better target populations at risk and ultimately improve child health outcomes.

## Methods

### Study population and outcomes data

We performed a retrospective secondary analysis on an existing database used to derive the Febrile Infants Risk Score at Triage (FIRST) (10). This prospective study included febrile infants ≤3 months old who presented to KK Women's and Children's Hospital ED between December 2017 and December 2021. Our institution is the larger of two tertiary paediatric EDs in Singapore, with an annual attendance of about 150,000 children. Fever was defined as an axillary or rectal temperature of at least 38 °C, as measured by the triage nurse while the infant was wrapped in a single layer of clothing. Infants with a recent history of fever at home prior to presentation but who were afebrile during triage assessment were not included.

### Study setting

Singapore is a multi-ethnic and multicultural city state with an estimated population size of 5.45 million as of June 2021 (11). The three major ethnic groups are Chinese, Malay, and Indian with a smaller group designated as “Others” encompassing a diverse mix of Caucasian, Eurasian, Filipino, among others. The multi-ethnic and communal living arrangements in Singapore provide a unique context for studying health outcomes across different socioeconomic strata.

### Socioeconomic Status measure: the Singapore Housing Index

In Singapore, one of the important markers of SES is home ownership, with 88.9% rates of home ownership in 2021 (12). Approximately 80% live in government subsidised Housing and Development Board (HDB) flats in 2021, with tiered subsidies available (13). Flat purchase prices are directly proportional to the number of rooms in the flat. Heavily subsidised rental housing, mostly 1- and 2-room flats, are also available for those who do not own homes, and this is subject to financial means testing.

The Singapore Housing Index (SHI) is a marker for SES which has been used to answer multiple health-related research questions in Singapore (14–19). Postal codes, unique to individual building, were used to determine SHI based on the weighted average number of rooms per unit for each building. It is a building-level marker that takes a value from 1 to 7 (with 7 indicating highest SES), and SHI has been shown to have a strong association with income and residence value (20)⁠.

Using previously described methods, we used open government data to ascertain SHI (20). Postal codes were obtained from electronic medical records. HDB dwellings were assigned an SHI value of 1–6 based on the average number of rooms per unit; those in private housing such as apartments, terrace houses, semi-detached houses, and condominiums were assigned a SHI value of 7. SHI was categorised into three groups, low (<3), medium (3–4.9), and high (>=5) as previously described and validated (14, 21). The categorisation is clinically meaningful as there is a proportional relationship between SHI and average household income: those assigned low SHI have an average household income of 2,997 SGD/month, while those assigned medium SHI have an average household income of 6,442–9,414 SGD/month, and those assigned high SHI have an average household income >12,723 SGD/month in year 2020 (21). The categorisation does not assume a normal distribution. The categories are treated as a categorical variable, with low SHI set as the reference category.

### Data and outcome variables

Study team members were trained to review the electronic health records. Data points were recorded into a spreadsheet and data verification was performed by one of the senior team members. We documented demographic information of patients, including age, ethnicity, birth gestation, gender, and residential postal code. Neonates were defined as infants <28 days old as per WHO definition (22). We excluded infants with birth gestation less than 35 weeks and defined late prematurity as those with birth gestation between 35 + 0 and 36 + 6 weeks. Routine triage data, including vital signs such as temperature, heart rate, respiratory rate, and oxygen saturations were collected. Severity index score (SIS) was defined as a composite measure of respiratory effort, colour, activity, temperature, and play. The SIS is used to assess the acuity of a patient, with SIS 10 signifying not very sick, SIS 8 or 9 moderately sick, and SIS 7 or less very sick (23). We also collected information on the presence of comorbidities, duration of fever, and maternal Group B streptococcus (GBS) status. Laboratory investigations included haemoglobin, total white blood cell count, absolute neutrophil count (ANC), C-Reactive Protein (CRP), and procalcitonin. Urine was assessed for leukocyte esterase (graded as negative, 1+, 2+, or 3+) and nitrite (positive or negative) with a colorimetric analyser system. CSF was analysed for cell count and clarity. Culture results from blood, urine, and CSF were obtained.

The primary outcome was the presence of SBI defined as bacteraemia, meningitis, and urinary tract infections (UTIs) (24). Invasive Bacterial Infection (IBI) is a subset of SBI that is defined as bacteraemia and meningitis (24). Diagnoses of bacteraemia and meningitis required the growth of pathogen in blood and CSF bacterial cultures, respectively. Cases of concurrent positive blood and urine cultures were coded as IBI. There were no cases of concurrent positive urine and CSF cultures. UTIs were defined as the presence of single or dual pathogen growth with >100,000 colony forming units (CFU)/mL in a clean catch specimen, or >10,000 CFU/mL in a catheterised specimen and with an abnormal urinalysis (positive for pyuria defined by ≥10 white blood cells/µL or positive for either leucocyte esterase or nitrite) (25). The secondary outcome was a composite of severe clinical outcomes, namely the need for fluid bolus resuscitation or inotropic support, emergent intravenous antibiotics (administered in the Emergency Department), or ventilator support (both invasive and non-invasive), and/or admission to High Dependency (HD) or Intensive Care Unit (ICU) care. We chose these clinical interventions because they are routinely reported for critically ill infants (at risk of sepsis and deterioration) who require more intense resource allocation.

### Statistical analysis

Categorical variables were described using frequencies and percentages while continuous variables were described using mean and standard deviation (SD) or median and interquartile range (IQR), depending on normality. ANOVA was used to compare means, and chi-square test was used to compare proportions. Fisher's exact test was used to compare IBI proportions specifically as sample size was small. Univariate and multivariable logistic regression analyses were performed for primary outcome of SBI, adjusting for SHI groups, whether the patient was a neonate (age <28 days), male gender, ethnicity, and late prematurity. Variable selection was based on existing literature, prior studies and univariate significance (10, 26). Clinical severity markers were not included in regression analysis as the aim was to assess baseline SES association. Additionally, logistic univariate and multivariable regressions were performed to predict the composite of severe clinical outcomes.

Unadjusted and adjusted odds ratios (aORs) with their corresponding 95% confidence intervals (95%CI) were presented. Level of significance of 0.05 was used. Statistical analyses were performed using R Statistical Software, v 4.0.5 (R Foundation for Statistical Computing).

### Ethics

Ethics approval was obtained for the primary study from the SingHealth Institutional Review Board E in Singapore (2023-2640) with waiver of informed consent. The procedures in this study adhered to the tenets of the Declaration of Helsinki.

## Results

### Baseline characteristics

**Figure 1:**
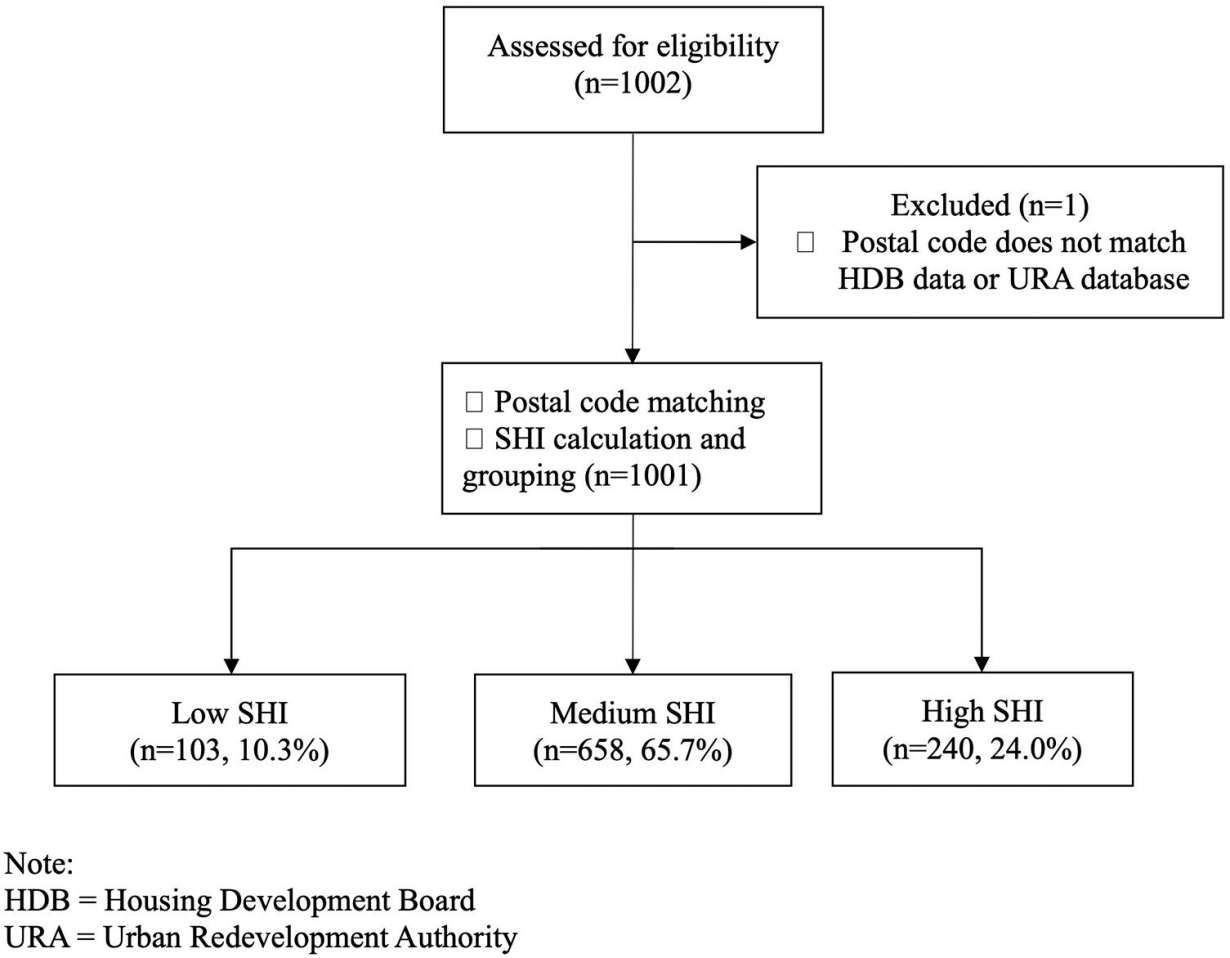
Flowchart of eligible patients who were analysed.

Among 1,002 cases in the database for febrile infants, we matched postal codes to SHI for 1,001 cases. There was one case where the postal code did not match either HDB data or Urban Redevelopment Authority (URA) private residences database. This case was excluded from further analysis (Figure 1).

Among 1,001 febrile infants analysed, the median age was 32 days (IQR 10–60), 44.5% were neonates, 57.2% were male, and 24.2% had positive history of maternal GBS colonisation (Table 1). At presentation, the mean temperature was 38.5 °C (SD 0.6), mean respiratory rate was 42/minute (SD 6), and mean Severity Index Score (SIS) was 9 (SD 0.8). The duration of fever was similar across groups. The prevalence of SBI amongst these infants was 17.6% (176/1,001). The SBI proportions in low, medium and high SHI groups were 12.6% (13/103), 17.5% (115/658), and 20% (48/240), respectively (*p* = 0.256).

**Table 1:** Patient demographics and clinical management, stratified by Singapore Housing Index (SHI) groups.

Variable		Overall	Low SHI	Medium SHI	High SHI	*p* value
		(*n* = 1,001)	(*n* = 103)	(*n* = 658)	(*n* = 240)	
Age in days, median (IQR)		32 (10, 60)	34 (15, 55)	31 (10, 59)	37 (10.75, 64)	0.241
Neonates (age <28 days) (%)		445 (44.5)	39 (37.9)	303 (46.0)	103 (42.9)	0.257
Male sex (%)		573 (57.2)	56 (54.4)	375 (57.0)	142 (59.2)	0.695
Ethnicity (%)	Chinese	534 (53.3)	31 (30.1)	367 (55.8)	136 (56.7)	<0.001
	Indian	89 (8.9)	9 (8.7)	52 (7.9)	28 (11.7)	
	Malay	286 (28.6)	56 (54.4)	209 (31.8)	21 (8.8)	
	Others	92 (9.2)	7 (6.8)	30 (4.6)	55 (22.9)	
Maternal GBS present (%)	Absent/Unknown	759 (75.8)	73 (70.9)	495 (75.2)	191 (79.6)	0.187
	Present	242 (24.2)	30 (29.1)	163 (24.8)	49 (20.4)	
Days of fever		1.2 (0.48)	1.2 (0.46)	1.2 (0.49)	1.1 (0.48)	0.862
Temperature in °C		38.5 (0.6)	38.5 (0.6)	38.4 (0.6)	38.5 (0.6)	0.408
Heart rate, per minute		162 (21)	164 (17)	161 (22)	162 (21)	0.583
Respiratory rate, per minute		42 (6)	42 (6)	42 (6)	42 (6)	0.818
Severity Index Score		9 (0.8)	9 (0.7)	9 (0.8)	9 (0.8)	0.449
Serious bacterial infections (%)		176 (17.6)	13 (12.6)	115 (17.5)	48 (20.0)	0.256
UTI (%)		152 (15.2)	10 (9.7)	106 (16.1)	36 (15.4)	0.242
IBI (%)		23 (2.3)	3 (2.9)	9 (1.4)	11 (4.6)	0.006
C-Reactive Protein		*n* = 916	*n* = 95	*n* = 606	*n* = 215	0.728
		17.4 (35.8)	20.2 (32.9)	17.1 (37.1)	17.1 (33.1)	
Procalcitonin, median (IQR)		*n* = 579	*n* = 58	*n* = 377	*n* = 144	0.725
		0.10 (0.05, 0.24)	0.11 (0.05, 0.21)	0.09 (0.05, 0.24)	0.10 (0.05, 0.32)	
White blood cell		*n* = 927	*n* = 97	*n* = 614	*n* = 216	0.702
		12.82 (5.29)	13.15 (5.91)	12.73 (5.95)	12.95 (5.68)	
Absolute neutrophil count		*n* = 927	*n* = 97	*n* = 614	*n* = 216	0.496
		5.66 (3.74)	5.64 (4.45)	5.57 (3.51)	5.92 (4.03)	
Fluid bolus required (%)		51 (5.1)	2 (1.9)	33 (5.0)	16 (6.7)	0.187
IV antibiotics given (%)		672 (67.1)	71 (68.9)	442 (67.2)	159 (66.2)	0.889
Lumbar puncture done (%)		476 (47.6)	48 (46.6)	313 (47.6)	115 (47.9)	0.975
Follow up given (%)		482 (48.2)	50 (48.5)	313 (47.6)	119 (49.6)	0.864
Length of hospital stay in days		4.1 (3.79)	4.2 (3.08)	3.9 (3.22)	4.5 (5.22)	0.134

IQR, interquartile range, GBS, group B Streptococcus, UTI, urinary tract infection, IBI, invasive bacterial infection, IV, intravenous.

### Invasive bacterial infections

Among 23 infants with invasive bacterial infections (IBI), there were 3 infants (2.9%) in low SHI (all concurrent bacteraemia with UTI), 9 (1.4%) in medium SHI (1 meningitis and 8 bacteraemia, out of which 3 were concurrent bacteraemia with UTI), and 11 (5.0%) in high SHI (4 meningitis, out of which 3 were concurrent bacteraemia and meningitis; and 7 bacteraemia, out of which 2 were concurrent bacteraemia with UTI) (*p* = 0.006).

There was no statistically significant difference of UTI proportions between low, medium, and high SHI groups (*p* = 0.242). Out of 152 cases of UTI, the most common pathogen grown in urine culture was *Escherichia coli* at 82.2% (125/152), followed by *Klebsiella pneumoniae* at 8.5% (13/152). Out of 18 cases of bacteraemia, *Escherichia coli* was found in 6 patients (33.3%) and group B *Streptococcus* in another 6 patients (33.3%). For cases of meningitis, the most common pathogen was *Streptococcus agalactiae* (group B *Streptococcus*) at 50% (3/6), and other pathogens identified were *Streptococcus gallolyticus*, *E. coli*, and *Enterobacter cloaca.* (Supplementary Table S1A–C).

### Regression analysis

After adjusting for male sex, neonate status, ethnicity, and late prematurity, neither high (aOR 1.714, 95% CI 0.844–3.480, *p* = 0.136) nor medium SHI (aOR 1.572, 95% CI 0.826–2.990, *p* = 0.168) was associated with lower odds of SBI, when compared to low SHI (Table 2).

**Table 2:** Associations between serious bacterial infections (SBIs) and risk factors in febrile infants.

Variables	Univariate Analysis		Multivariable Analysis	
	Odds ratio (95% CI)	*p*	Adjusted Odds ratio (95% CI)	*p*
SHI				
Low	1.00 (Ref)		1.00 (Ref)	
Medium	1.466 (0.792–2.713)	0.223	1.572 (0.826–2.990)	0.168
High	1.731 (0.893–3.355)	0.104	1.714 (0.844–3.480)	0.136
Neonate (age <28 days)	0.272 (0.185–0.401)	< 0.001	0.270 (0.182–0.400)	< 0.001
Male	2.277 (1.591–3.260)	< 0.001	2.227 (1.547–3.251)	< 0.001
Ethnicity				
Chinese	1.00 (Ref)		1.00 (Ref)	
Malay	0.604 (0.405–0.903)	0.014	0.685 (0.447–1.050)	0.082
Indian	0.736 (0.401–1.353)	0.324	0.744 (0.395–1.402)	0.361
Others	0.830 (0.465–1.482)	0.529	0.881 (0.473–1.639)	0.688
Late Prematurity	0.434 (0.185–1.021)	0.056	0.414 (0.172–0.995)	0.049

SHI, Singapore Housing Index, CI, Confidence Interval.

In terms of secondary outcomes, no evidence of significant difference was found in the length of hospital stay (mean ranging: 3.9–4.5 days, *p* = 0.134). There was also no evidence of significant differences in the need for fluid bolus resuscitation (*p* = 0.187) or need for intravenous antibiotics (*p* = 0.889). Four cases required ICU admission. Only one child required inotropic support due to a diagnosis of bacteraemia with meningitis and subdural empyema requiring craniotomy and evacuation. Three patients required non-invasive ventilatory support, with none requiring invasive ventilatory support. After adjustment, neither high nor medium SHI was associated with lower odds of severe outcomes, when compared to low SHI (Table 3).

**Table 3:** Associations between severe clinical outcomes^a^ and risk factors in febrile infants.

Variables	Univariate Analysis		Multivariable Analysis	
	Odds ratio (95% CI)	*p*	Adjusted Odds ratio (95% CI)	*p*
SHI				
Low	1.00 (Ref)		1.00 (Ref)	
Medium	1.816 (0.547–6.025)	0.329	1.584 (0.468–5.367)	0.460
High	2.381 (0.678–8.356)	0.176	2.038 (0.555–7.483)	0.283
Neonate (age <28 days)	0.348 (0.181–0.671)	0.002	0.354 (0.183–0.686)	0.002
Male	1.056 (0.602–1.850)	0.850	0.992 (0.561–1.756)	0.979
Ethnicity				
Chinese	1.00 (Ref)		1.00 (Ref)	
Malay	0.460 (0.226–0.936)	0.032	0.496 (0.237–1.037)	0.062
Indian	0.144 (0.020–1.063)	0.057	0.151 (0.020–1.122)	0.065
Others	0.428 (0.129–1.414)	0.164	0.419 (0.123–1.426)	0.164
Late Prematurity	1.824 (0.751–4.433)	0.184	1.874 (0.752–4.666)	0.177

^a^ Defined by either fluid bolus resuscitation or inotropic support or emergent intravenous antibiotics or ventilator support or admission to high dependency (HD) or intensive care unit (ICU).

SHI, Singapore Housing Index, CI, Confidence Interval.

Based on census data, we report the geospatial distribution of febrile infant cases in our database per 1,000 0–4-year-olds in Supplementary Figure S1.

## Discussion

In Singapore, low SHI among adult patients has been reported to be associated with poor health outcomes across various illnesses (14–19). Evidence in the paediatric population remains scarce. A retrospective cross-sectional study performed among febrile infants in USA reported that SBI risk was significantly higher in neighbourhoods with high rates of childhood poverty compared to more affluent neighbourhoods (6). Interestingly, in our study, we found no strong evidence to suggest that SBI prevalence was higher among infants with low SHI presenting to the ED.

In our setting, there is differential access to healthcare between the various socioeconomic groups. Public hospitals, which accounts for 80% of inpatient beds, primarily serve lower SES patients. In contrast, private hospitals, with 20% of inpatient beds, are more accessible to higher SES patients and those with private insurance (30, 31). Higher SES patients may preferentially seek care in the private sector. However, public hospitals are equipped with subspecialty support and generally cater to more complex cases. In our study, it is plausible that febrile infants from the higher SHI group with self-resolving fevers were managed at private hospitals or clinics, with more severe cases referred to public hospitals. Conversely, febrile infants from the lower SHI group are less likely to utilise private sector services due to financial reasons, explaining differences in healthcare use.

Additionally, we postulate that our findings could be attributed to the unique characteristics of Singapore, an urbanised Asian country. Singapore is a small country with a total land area of approximately 734.3 km^2^ (27). Singapore has one of the lowest infant mortality rates in the world at 1.8 per 1,000 live births (28). Public housing in Singapore is not geographically segregated according to SES and the country's Urban Redevelopment Authority ensures equitable population distribution and resource allocation (29). This promotes equal access to healthcare facilities for all residents regardless of SES (29). As a result, this may mitigate SES related disparities in healthcare access observed elsewhere.

Furthermore, local initiatives such as KidSTART Programme identify at-risk families from the antenatal period and provide ongoing community support, home visits, and health screenings from birth through early childhood. These programs may effectively improve health literacy and educate low SES families to promptly seek medical care thus reducing SBI rates in this group (9). We postulate that equitable access to high-quality healthcare for all socioeconomic groups levelled the risk, and this may be investigated further in future studies.

As defined by Hahn, social determinants of health are the social systems and resources that society controls and distributes, which in turn affect health outcomes and demographic health trends (32). While SHI serves as a valuable SES surrogate, it is not comprehensive. Other factors such as educational level, income inequality, employment status, or requirement for financial assistance also play important roles (12). Therefore, SHI alone may not fully capture the social context of each family, nor encapsulate other social determinants of health.

UTI is the most common SBI, similar to a prior report (6). In our population, UTI was least prevalent in the low SHI group. At the same time, Malay ethnicity comprised 54.4% of the low SHI group, 31.8% of the medium SHI group, and 8.8% of the high SHI group. One of the protective factors against UTI is circumcision (33, 34). Majority of the Malay community are Muslims (35), and it is a common religious practice for Muslims to circumcise male infants. Since infants who are circumcised have a lower risk of developing UTI, this could account for a lower rate of UTI in the low SHI group. However, this is a hypothesis-generating observation and further data is required to confirm this finding as circumcision data were unavailable in our study.

We recognise the limitations of our study. This was a single-centre study involving a public tertiary hospital, and this setting may introduce selection bias as higher SES patients may prefer to use private healthcare facilities. Therefore, this study requires external validation before our findings can be generalised. Furthermore, this study reflected presentations to a single-centre ED and did not show population-level incidence. Additionally, we recognise that there are other potential confounders such as details on comorbidity and feeding practices that were not documented and could not be included in the adjustment. In future, a purposefully designed prospective study, with variables collected specifically to address SES and SBI risk, would be ideal to explore this topic robustly and confirm any associations suggested by this study.

Another limitation was that the incidence of IBI and severe clinical outcomes in our population were low, therefore we were not adequately powered to analyse secondary outcomes individually including length of ICU stay, need for ventilatory support and inotropic support. Due to the small number of IBI cases (*n* = 23), comparisons of IBI numbers between SHI groups is to be interpreted with caution. In addition, the number for composite of severe outcome was small at 53 cases, suggesting that this study is underpowered to investigate factors affecting secondary outcomes.

We were not able to account for the physical distance between patients' homes and our hospital, and how this affected health-seeking behaviours. In this study on association between SHI status and SBI prevalence, we limited our adjustment to patients' demographics (neonate status, sex, ethnicity and late prematurity). We recognise that future studies on individual infant's SBI risk should account for other maternal and infant risk factors for SBIs. We also recognise that SHI has limitations as a proxy for SES as it is derived from postal code and average room count per dwelling, and it may not fully reflect household SES. Nonetheless, to our knowledge, our study is one of the first in Asia to explore the associations between sociodemographic characteristics and SBI risk in a racially diverse paediatric population.

It is crucial that we understand the association between social determinants of health and clinical outcomes as findings can inform health policies. Further studies are needed to characterise the socioeconomic differences beyond housing, including household income, insurance coverage, accessibility to healthcare and at-risk families known to social services, as well as ethnicity. Further studies should also include both public and private hospitals and clinics to further elucidate the differential access between various SHI groups.

## Conclusion

In our study population, young infants from high SHI were not at lower risk for SBIs. Future studies should include purposefully designed prospective studies, with variables collected specifically to address SES and SBI risk to investigate the relationship between other social determinants of health and the prevalence and outcomes of young infant SBIs.

## Data Availability

The raw data supporting the conclusions of this article will be made available by the authors, without undue reservation.
