# Supplementary material for: Singapore Housing Index and prevalence of serious bacterial infections among febrile infants
Source: Front Pediatr. 2026 Mar 2;14:1716413. doi: 10.3389/fped.2026.1716413 (PMC12989585; doi:10.3389/fped.2026.1716413)

Supplementary tables and figure

Supplementary Table 1A. Pathogen grown in urine culture in cases of urinary tract infection

| Urine culture | Frequency | Percentage |
| --- | --- | --- |
| Escherichia coli | 110 | 72.37% |
| Escherichia coli (ESBL) | 1 | 0.66% |
| Escherichia coli & Klebsiella pneumoniae | 6 | 3.95% |
| Escherichia coli & Klebsiella aerogenes | 1 | 0.66% |
| Escherichia coli & Proteus mirabilis | 4 | 2.63% |
| Escherichia coli & Acinetobacter species | 1 | 0.66% |
| Escherichia coli & Enterococcus faecalis | 2 | 1.32% |
| Klebsiella pneumoniae | 12 | 7.89% |
| Klebsiella pneumoniae & Enterococcus faecalis | 1 | 0.66% |
| Klebsiella oxytoca & Citrobacter freundii | 1 | 0.66% |
| Klebsiella aerogenes | 3 | 1.97% |
| Citrobacter koseri | 3 | 1.97% |
| Enterococcus faecalis | 3 | 1.97% |
| Enterobacter aerogenes | 1 | 0.66% |
| Enterobacter cloacae | 1 | 0.66% |
| Enterobacter hormaechei | 1 | 0.66% |
| Pseudomonas aeruginosa | 1 | 0.66% |
| Total number | 152 |  |

Supplementary Table 1B. Pathogen grown in blood culture in cases of septicaemia

| Blood culture | Frequency | Percentage |
| --- | --- | --- |
| Streptococcus agalactiae (Group B) | 6 | 33.3% |
| Escherichia coli | 5 | 27.8% |
| Escherichia coli (ESBL) | 1 | 5.6% |
| Klebsiella pneumoniae | 2 | 11.1% |
| Staphylococcus aureus | 2 | 11.1% |
| Enterobacter cloacae complex | 1 | 5.6% |
| Enterococcus faecalis | 1 | 5.6% |
| Total number | 18 | 100.0% |
|  |  |  |

Supplementary Table 1C. Pathogen grown in cerebrospinal fluids (CSF) in cases of meningitis

| CSF Culture | Frequency | Percentage |
| --- | --- | --- |
| Streptococcus agalactiae (Group B) | 2 | 33.3% |
| Escherichia coli | 1 | 16.7% |
| Streptococcus agalactiae (Group B) | 1 | 16.7% |
| Streptococcus gallolyticus ssp pasteurianus | 1 | 16.7% |
| Enterobacter cloacae | 1 | 16.7% |
| Total number | 6 | 100.0% |

Supplementary figure 1. Febrile infants per thousand 0-4 year old by planning areas.


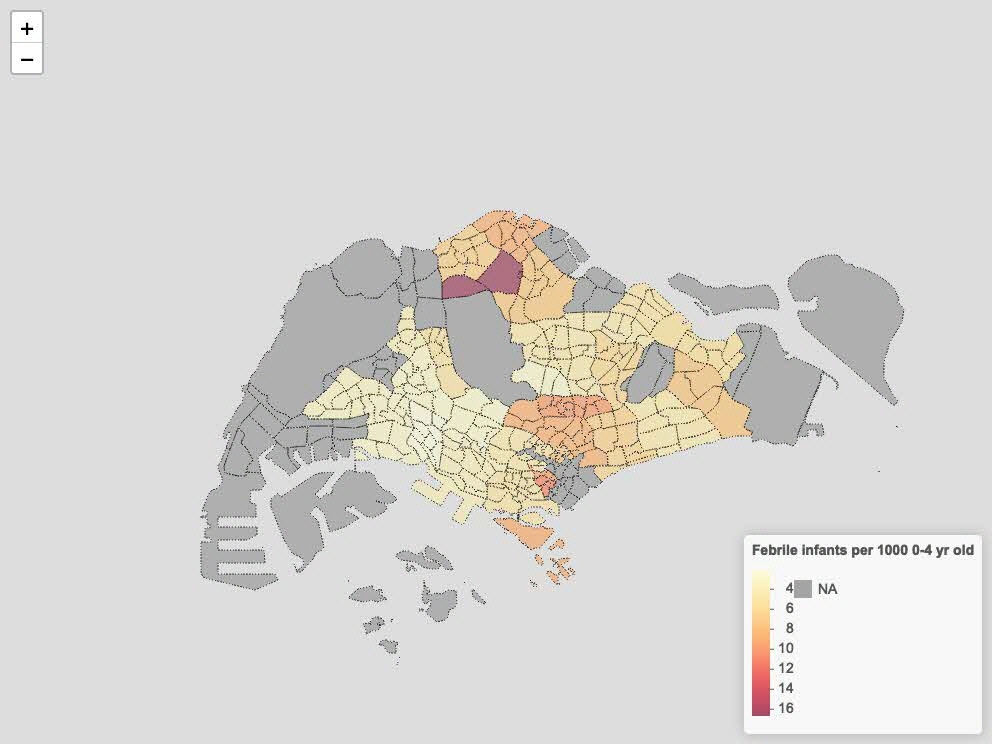

Supplement: Supplementary file 1 [file Supplementaryfile1.docx]
